# Correction: Single-cell analysis of mosquito hemocytes identifies signatures of immune cell subtypes and cell differentiation

**DOI:** 10.7554/eLife.85158

**Published:** 2022-11-28

**Authors:** Hyeogsun Kwon, Mubasher Mohammed, Oscar Franzén, Johan Ankarklev, Ryan Smith

**Keywords:** Other

Kwon H, Mohammed M, Franzén O, Ankarklev J, Smith RC. 2021. Single-cell analysis of mosquito hemocytes identifies signatures of immune cell subtypes and cell differentiation. *eLife* **10**:e66192. doi: 10.7554/eLife.66192.

Published 28 June 2021

After further review and use of the marker enrichment originally published in Supplementary file 3, we felt that it did not accurately reflect our data. This analysis was performed using the Seurat function “FindAllMarkers” in November of 2020 (Seurat version 3.1.5) and seems to be the source of this reproducibility issue. When our data set was again examined using version 4.1.1 of the Seurat package, we see dramatically different results using this improved software version that are more consistent and reflective of the enriched/depleted markers representing the individual cell clusters in our data set. As a result, the corrected “FindAllMarkers” analysis from version 4.1.1 are now reflected in the updated Supplementary file 3.

This error did not affect any results or conclusions of the paper.

The article has been corrected accordingly.

