# Supplementary material for: Single-cell analysis of mosquito hemocytes identifies signatures of immune cell subtypes and cell differentiation
Source: eLife. 2021 Jul 28;10:e66192. doi: 10.7554/eLife.66192 (PMC8376254; doi:10.7554/eLife.66192)
Supplement: Supplementary file 6. [file elife-66192-supp6.docx]

**Table S6. Primers for qRT-PCR analysis**

**Primer Gene ID Sequence (5’- 3’)**

LRIM15-F AGAP007045 CGATCCTGATCCTGAACGTGGGCTTC

LRIM15-R GCAAGCAAGCCACTCACAAATCCTCG

LRIM16A-F AGAP028028 ATCAGAGTGCAGCACAAGTTGAAGGT

LRIM16A-R TCTCTGTTAGCATAGCGCCTTCGTTC

Lz-F AGAP002506 GCACCGTCAATCAGAACCAA

LZ-R TGCCACTGATCGAATGCTTG

NimB2-F AGAP029054 CAATCTGCTCAAATGGCTGCTTCCACG

NimB2-R GCTGCAAACATTCGGTCCAGTGCATTC

PPO1-F AGAP002825 GACTCTACCCGGATCGGAAG

PPO1-R ACTACCGTGATCGACTGGAC

PPO2-F AGAP006258 TTGCGATGGTGACCGATTTC

PPO2-R CGACGGTCCGGATACTTCTT

PPO3-F AGAP004975 CTATTCGCCATGATCTCCAACTACG

PPO3-R ATGACAGTGTTGGTGAAACGGATCT

PPO4-F AGAP004981 GCTACATACACGATCCGGACAACTC

PPO4-R CCACATCGTTAAATGCTAGCTCCTG

PPO5-F AGAP012616 GTTCTCCTGTCGCTATCCGA

PPO5-R CATTCGTCGCTTGAGCGTAT

PPO6-F AGAP004977 GCAGCGGTCACAGATTGATT

PPO6-R GCTCCGGTAGTGTTGTTCAC

PPO8-F AGAP004976 CCTTTGGTAACGTGGAGCAG

PPO8-R CTTCAAACCGCGAGACCATT

PPO9-F AGAP004978 TGTATCCATCTCGGACGCAA

PPO9-R AAGGTTGCCAACACGTTACC

rpS7-F AGAP010592 ACCCCATCGAACACAAAGTTGACACT

rpS7-R CTCCGATCTTTCACATTCCAGTAGCAC

SCRB3-F AGAP005725 CATCGGGACAGCTACATCCT

SCRB3-R TTATTGCTGCTACCGTTGCC

SCRB9-F AGAP004846 CGATATTCGGCGATGCAACT

SCRB9-R CACGCATGACACGATTCAGT
