# Supplementary material for: Single-cell analysis of mosquito hemocytes identifies signatures of immune cell subtypes and cell differentiation
Source: eLife. 2021 Jul 28;10:e66192. doi: 10.7554/eLife.66192 (PMC8376254; doi:10.7554/eLife.66192)
Supplement: Supplementary file 7. [file elife-66192-supp7.docx]

**Table S7. Primers for RNAi**

**Primer Gene ID Sequence (5’- 3’)**

T7-GFP F TAATACGACTCACTATAGGGAGAATGGTGAGCAAGGGCGAGGAGCTGT

T7-GFP R TAATACGACTCACTATAGGGAGATTACTTGTACAGCTCGTCCATGCC

T7-Lz F AGAP002506 TAATACGACTCACTATAGGGCTGCAACCGTCCCAGAACAACGGC

T7-Lz R TAATACGACTCACTATAGGGACAAACCGGAGATCGTTGAATTTGG
